# JTM advances in uncharted territories: diseases and disorders of unknown etiology

**DOI:** 10.1186/s12967-017-1293-6

**Published:** 2017-09-13

**Authors:** Monica C. Panelli

**Affiliations:** University of Pittsburgh Cancer Institute, Pittsburgh, USA

**Keywords:** Etiology, Aetiology, Myalgic Encephalomyelitis, Chronic Fatigue Syndrome, Fibromyalgia Syndrome

## Abstract

We are delighted to announce a new section in the *Journal of Translational Medicine*, ‘Illnesses of Unknown Etiology’. This section aims to provide a translational medicine forum for the publication of research on illnesses, multisystem diseases and syndromes of unknown etiology. Examples of these include Myalgic Encephalomyelitis/Chronic Fatigue Syndrome and Fibromyalgia Syndrome.

In this Editorial we announce a new section in the *Journal of Translational Medicine*: Illnesses of Unknown Etiology. This section is dedicated to the dissemination of research on diseases of unknown etiology such as Myalgic Encephalomyelitis (ME)/Chronic Fatigue Syndrome (CFS). The core objective of this section of the *Journal of Translational Medicine* is to welcome novel diagnostic criteria, tools, and biomarkers of disease, as well as modalities that may reduce or alleviate some of the multiple symptoms characterizing these diseases.

The focus on multisystem diseases of unknown cause highlights not only the immediate need in medicine to research the origin of an unknown disease with multiple symptoms, but also the necessity to find novel ways to diagnose and discriminate diseases that have no clear etiology but represent a dramatic reality affecting large populations of patients.

According to the Centers for Disease Control and Prevention (CDC) one million Americans have CFS and 0.4% of people worldwide suffer from ME/CFS [1]. ME/CFS is a complex, multifaceted illness, that lacks a universally accepted case definition with debilitating and serious health problems characterized by persistent, medically unexplained fatigue, and symptoms such as musculoskeletal pain, sleep disturbance, headaches and impaired concentration and short-term memory [2, 3]. The World Health Organization (WHO) has classified ME/CFS as a neurological disorder [4]. At least one-quarter of individuals with ME/CFS are bedbound or housebound at some point in the illness and most never regain their pre-disease level of functioning. ME/CFS strikes people of all ages and racial, ethnic, and socioeconomic groups, and is diagnosed most frequently in women [3]. A distinctive characteristic of the illness is post-exertion malaise, which requires an extended recovery period [3].

The economic burden of ME/CFS, including annual health care costs, is estimated to be between $1.9 billion and $7.2 billion [5].

The complexity of this illness characterized by a plethora of symptoms inevitably calls for the expertise of researchers in very different fields of Medicine. A working group has been established to research on the cause, prevention diagnosis, pathophysiology and treatment of ME/CFS (Trans NIH/ME/CFS working group) [3] in the USA.

Numerous clinical trials and studies have currently (7/18/2017) been completed (18/39), are ongoing (2/39) or are recruiting (7 of 39) in the US [6, 7] and around the world (The European ME/CFS Biomarker Landscape project-an initiative of the European network EUROMENE) [8]. These trials aim to closely examine and research the clinical and biological characteristics of this disease, with the intention of improving our understanding of its cause and progression.

In various areas of research there has been progress in understanding ME/CFS biomarkers and symptoms [9]. Diagnostic biomarkers for this disease such as Activin B [10] have been reported recently. Stringer and Skowera [11, 12] have demonstrated the efficacy of cytokine profiling as biomarkers of disease, particularly multiplex panels of inflammatory cytokines. The emerging causative role of autoimmunity [13], immune-inflammatory, oxidative and nitrosative stress (O&NS) pathways [14] are starting to be elucidated. Interestingly, Falkenberg et al. [15], reported acute psychosocial stress-mediated changes in the expression and methylation of perforin as an indication of dysregulation of immune responses in ME/CFS patients. A distinct phenotype of T and NK cells [16], as well as downregulation of immune responses by TGFb in NK cell, were also shown to be linked to immune dysfunction in ME/CFS cohorts [17–19].

There is evidence that immune-inflammatory and oxidative and nitrosative stress (O&NS) pathways play a role in the pathophysiology of ME/CFS [14] There is also evidence that these neuroimmune diseases are accompanied by hypothalamic–pituitary–adrenal (HPA) axis hypoactivity, as indicated by lowered baseline glucocorticoid levels [14]. Findings show that aActivation of immune-inflammatory and oxidative and nitrosative stress (O&NS) pathways in ME/CFS are probably not secondary to HPA axis hypoactivity and activation of these pathways may underpin HPA axis hypofunction in ME/CFS. Mechanistic explanations comprise increased levels of tumor necrosis factor-α, T regulatory responses with elevated levels of interleukin-10 and transforming growth factor-β, elevated levels of nitric oxide, and viral/bacterial-mediated mechanisms. HPA axis hypoactivity in ME/CFS is most likely a consequence and not a cause of a wide variety of activated immune-inflammatory and O&NS pathways in ME/CFS [14].

Most recently, Glassford [4] summarized the processes that collectively enhance the potential for multi-systemic disarray of ME/CFS, reporting endocrine pathway aberration, immune and mitochondrial dysfunction, neurodegeneration, intractable synergistic neuro-glial dysfunction (gliopathy), autoimmunity, and central neuronal sensitization.

According to the Arthritis foundation [20], research shows that 50–70% of people with a diagnosis of either ME/CSF or Fibromyalgia Syndrome (FMS) also fit the criteria of the other illness. While in 2015 [1] ME/CFS was legitimized as a disease instead of a syndrome, fibromyalgia still remains characterized as a syndrome (FMS). In addition to the symptoms shared with patients with MS/CFS, people with FMS often present with symptoms of abnormal pain perception processing [1, 21]. FMS affects about 4 million US adults, about 2% of the adult population. The cause of fibromyalgia is not known, but it can often be effectively treated and managed. The first drug for FMS (pregabalin, Lyrica) has been approved in 2007. In comparison, no FDA approved modality exists to date for ME/CFS.

This section of *Journal of Translational Medicine* intends to be an international and multidisciplinary platform of scientific exchange and interaction among the various medical fields involved in diagnosing and treating/managing the symptoms of patients with ME/CFS and diseases of unknown etiology. The expectation is that this effort will not only accelerate the ongoing progress in this area of research, but first and foremost help improve the number of patients receiving the correct diagnosis and targeted medical care for their disease. This is also in line with the broader-spectrum objective of the *Journal of Translational Medicine* of spanning its publications across all areas of medicine.

As we inaugurate this new section in *Journal of Translational Medicine* on Illnesses of Unknown Etiology, we invite interested scientists to submit their work for publication.
